# Effects of L-Glutamine Supplementation during the Gestation of Gilts and Sows on the Offspring Development in a Traditional Swine Breed

**DOI:** 10.3390/ani11030903

**Published:** 2021-03-22

**Authors:** Marta Vázquez-Gómez, Consolación García-Contreras, Susana Astiz, Laura Torres-Rovira, José Luis Pesantez-Pacheco, Ana Heras-Molina, Teresa Castro Madrigal, Clemente López-Bote, Cristina Óvilo, Antonio González-Bulnes, Beatriz Isabel

**Affiliations:** 1 Faculty of Veterinary Medicine, UCM, Av. Puerta de Hierro s/n, 28040 Madrid, Spain; martavazgomez@gmail.com (M.V.-G.); tcastro@ucm.es (T.C.M.); clemente@ucm.es (C.L.-B.); 2 Facultat de Veterinària, Universitat Autònoma de Barcelona, Edifici V, Trav. dels Turons, 08193 Bellaterra, Spain; 3 SGIT-INIA, Ctra. De La Coruña Km. 7.5, 28040 Madrid, Spain; congarcon@gmail.com (C.G.-C.); astiz.susana@inia.es (S.A.); torrerovi@gmail.com (L.T.-R.); jose.pesantez@ucuenca.edu.ec (J.L.P.-P.); delasheras.ana@inia.es (A.H.-M.); ovilo@inia.es (C.Ó.); bulnes@inia.es (A.G.-B.); 4 School of Veterinary Medicine and Zootechnics, Faculty of Agricultural Sciences, University of Cuenca, Avda. Doce de Octubre, 010220 Cuenca, Ecuador; 5 Faculty of Veterinary Sciences, Universidad Cardenal Herrera-CEU, CEU Universities, C/Tirant lo Blanc, 7, Alfara del Patriarca, 46115 Valencia, Spain

**Keywords:** amino acids, fatty acids, mTOR, parity, pig, pregnancy

## Abstract

**Simple Summary:**

Nutritional strategies during pregnancy in swine production are considered essential to increase the number of piglets born alive and improve their survival and development. Amino acids, such as glutamine, are among the best compound to introduce in commercial farms after obtaining positive results in trials carried out in selected swine breeds. However, several critical productive factors have to be assessed before translating these strategies to the farm level to ensure the best balance between benefits and investments. The current study focused on the effects of prenatal L-glutamine supplementation on the offspring of Iberian gilts and sows under farm conditions. It is the first trial of amino acid supplementation during pregnancy carried out in traditional swine breeds. These non-selected swine breeds show productive or physiological differences that could affect the supplementation effect. Indeed, although there were changes at the molecular and tissue level, these effects did not turn into advantageous effects for the offspring of traditional breeds. The present study shows the importance of pre-testing nutritional strategies under the final conditions and breeds of implementation and the need to deepen at the molecular level to improve the biological interpretation of findings.

**Abstract:**

The use of amino acids during pregnancy, such as glutamine (Gln), seems to be a promising strategy in selected swine breeds to improve the offspring prenatal development. The main goal of the current study was to assess the development of the offspring from parity 1–3 sows of a traditional breed, which were supplemented with 1% glutamine after Day 35 of gestation, under farm conditions. A total of 486 (288 treated) piglets from 78 (46 treated) Iberian sows were used. At birth and slaughterhouse, fatty acid composition, metabolism, and mTOR pathway gene expression were analyzed. At birth, treated newborns showed greater amounts of specific amino acids in plasma, such as glutamine, asparagine, or alanine, and Σn-3 fatty acids in cellular membranes than control newborns. The expression of genes belonging to mTOR Complex 1 was also higher in treated piglets with normal birth-weight. However, these findings did not improve productive traits at birth or following periods in litters from supplemented gilts (parity 1) or sows (parities 2–3). Thus, further research is needed to properly understand the effects of prenatal glutamine supplementation, particularly in traditional swine breeds.

## 1. Introduction

Swine production is facing new challenges to improve its efficiency. One of the current objectives is to improve prolificacy without penalizing the offspring survival and development. The possible damaging effect of the intrauterine growth restriction process (IUGR) on the offspring, mainly linked to large litters, is well-known, as well as the negative consequences on their postnatal development [1,2,3,4,5]. Many studies on selected swine breeds have shown the IUGR impact on pig production by inducing lower homogeneity and low birth-weight (BIW) pigs [6,7,8]. These effects have also been described in traditional swine breeds, even with a greater impact than in modern breeds [9,10]. Although traditional breeds show lower reproductive parameters [11,12,13,14], the increase in litter size results in lower BIW and higher variability than in selected swine breeds [10]. Even the delay of days to market is longer in traditional breeds [10]. This consequence affects farm profitability and makes clear the wide range of improvement to work on useful strategies, particularly in traditional and fatty breeds, which is a growing industry due to its high-quality cured pork products.

At the sow level, the nutritional strategies are essential to diminish negative consequences in piglets throughout their productive lifetime. Among the prenatal nutritional strategies, amino acids (AA) clearly stand out. Particularly some functional AA, such as arginine [Arg], glutamine [Gln, AA abbreviation], and proline [Pro], have been the target of the largest number of studies on pregnancy in selected swine breeds [15,16,17,18,19,20]. They have shown benefits such as increased fetal-placental unit growth and reduced variation in BIW, with Arg being the most commonly AA used in previous studies [15,19,21,22,23,24,25,26,27]. Glutamine is particularly interested because it is one of the most abundant AA in fetal tissue protein and is a primary energy source for the fetal small intestine [18,19]. However, there remain significant factors to be assessed before translating this knowledge into farm strategy to guarantee the best equilibrium between advantages and investments. These factors include the parity number (Pa), the supplementation period, and the current farm conditions, particularly for Gln. Moreover, previous studies were only developed in selected swine breeds. There are no studies on the effect of the prenatal supplement of AA on traditional breeds, although their metabolic differences could produce differences in the response [28,29,30,31,32,33,34].

Hence, the present study aimed at determining the effects of prenatal Gln supplementation on the prenatal and postnatal developments of the offspring and their carcass and meat quality under farm conditions in a traditional swine breed. The second objective was to examine the interaction of prenatal Gln supplementation and the parity of sow.

## 2. Materials and Methods

### 2.1. Animals and Design

The experiment was carried out in a commercial farm, Ibéricos de Arauzo 2004 S.L. (Zorita de la Frontera, Salamanca, Spain), according to the European Union Directive and the Spanish Policy for Animal Protection RD53/2013. As a traditional breed, the Iberian pig was used in this trial. The management of sows and their offspring followed standard commercial farm practices, housing them indoors under controlled temperature, and electronic chip identification was used. Moreover, sows and offspring were fed with standard grain-based diets for Iberian pigs (diets shown in Table S1; [35]).

A total of 78 Iberian sows (Retinto variety), from parity (Pa) 1 to 3, were inseminated with cooled semen from Duroc PIC boars (Genus plc, Worcester, UK). Day 36 of pregnancy, 46 pregnant sows (Pa1: 21 gilts, Pa2: 16 sows, Pa3: 9 sows; treated group), randomly chosen, were supplemented with 1% L-glutamine (GLN [treated group abbreviation]; S.A. Ajinomoto OmniChem N.V., Wetteren, Belgium) on the gestation diet up to delivery. The 32 sows of the control group (C; Pa1: 10 gilts, Pa2: 13 sows, Pa3: 9 sows) were fed with the same diet without supplementation. Average daily feed intakes obtained during pregnancy, after Day 35, were between 1 and 1.5 kg with no difference by treatments. Sows were housed in groups from Day 35 to Day 101 of pregnancy and, afterward, individually allocated in pens until weaning. After birth, piglets were sexed and weighed, and a total of 486 alive piglets (C: 198, 53% males; GLN: 288, 49% males) were measured and allocated to mothers until weaning. Males were surgically castrated within the two first days of birth.

Piglets were classified into two birth-weight (BIW) categories, Low and Normal BIW (LBIW and NBIW), for experimental purposes. The classification cut-off value relied on previous studies on the same farm (BIW ≤ 0.99 kg; [9,10]). At weaning (average age, 24 days), 136 control (C; 51% males) and 151 treated (GLN; 50% males) piglets randomly selected were weighed and measured. Later, piglets were housed, distributed by treatment and sex in groups of 12 piglets/pen maximum. Pigs were monitored until slaughter. At 215 days-old, 96 control (37% male) and 103 treated pigs (47% males) were sampled. Finally, 54 control (61% males) and 79 treated pigs (62% males) were sampled at the slaughterhouse.

### 2.2. Birth Data and Offspring Development

Birth data were recorded per sow. The BIW mean and its SD and coefficient of variation (CoV) were calculated per sow using the total piglets born (without stillbirths) and statistically analyzed by parity and treatment. The remaining birth data were assessed based on piglets born alive.

Pigs were individually weighed at birth, weaning, and slaughterhouse. Pigs were shipped to the slaughterhouse in three batches. The first and second batches were slaughtered when pigs reached the minimum market carcass weight established for the Iberian breed (115 kg; Day 244 to 267 of average age). The remaining pigs were sent to market regardless of their body weight on Day 270 of average age. Average daily weight gain (ADWG) was individually calculated for the lactation phase, for the following period (growing and fattening) until the slaughterhouse and for the whole productive life.

At birth and weaning, morphological measurements (occipito-nasal length, biparietal diameter, trunk length, maximum thoracic diameter, and abdominal and thoracic circumferences) were recorded with a measuring tape. At weaning and 215 days-old, backfat thickness, distinguishing the inner and outer layers, and *longissimus dorsi* (LD, loin) muscle diameter were determined with ultrasound (SonoSite Inc., Bothell, WA, USA) at the P2 point (level of the head of the last rib). At the slaughterhouse, the length of carcasses (from the posterior edge of the *symphysis pubica* to the anterior edge of the first rib) and the backfat thickness (at the last rib) were measured with a tape. Carcass yield was calculated individually (carcass weight/body weight).

### 2.3. Tissue Sampling at Slaughter

At birth, 24 control and 24 treated piglets (6 LBIW and 6 NBIW piglets per sex) were euthanized and sampled. To avoid reducing the sample size at weaning, only eight control newborns (4 LBIW and 4 NBIW) were selected from control sows to slaughter at birth. The remaining euthanized control newborns came from sows with similar characteristics to our control group. Selected neonates were euthanized by stunning and exsanguination according to RD53/2013 standard procedures. Immediately head, carcass, and total and individual viscerae (brain, heart, intestine, kidneys, liver, lungs, pancreas, and spleen) were weighed. Later, weight-ratios of carcass and individual viscerae to BIW were calculated. Duodenal samples for gene expression analysis were immediately submerged in RNAlater (Invitrogen, Carlsbad, CA, USA) and stored at −20 °C. Samples of brain, the right lateral lobe of the liver, and LD muscle were also biobanked at −20 °C until fatty acids (FA) composition analysis.

At the slaughterhouse, samples of the right lateral lobe of the liver, LD muscle, and subcutaneous backfat fat at the measurement level (P2 point) were biobanked until FA composition analysis. On the same sampling day, a second sample of LD muscle was also used for the pH and drip-loss analyses [36]. Duodenal samples were also collected and stored at −80 °C.

### 2.4. Amino Acids, Glucose and Lipid Metabolism and Oxidative Status

Blood samples were drawn with vacuum tubes (Vacutainer Systems Europe, Meylan, France) from euthanized neonates at birth and after fasting at the slaughterhouse. Samples were centrifuged at 1500× *g* for 15 min, and plasma was separated and stored at −80 °C until analyses of AA and glucose (fructosamine) and lipid metabolism. The AA analysis of plasma samples was carried out by the Instrumental Techniques laboratory of the Universidad de Valladolid (UVA, Valladolid, Spain). It was performed using the ZORBAX Eclipse Plus method with an Agilent 1100 HPLC system [37]. Parameters of glucose and lipid (total cholesterol, high-density lipoprotein cholesterol [HDL-c], low-density lipoprotein cholesterol [LDL-c], and triglycerides) profiles were assessed by a clinical analyzer (Saturno 300 plus, Crony Instruments Srl, Rome, Italy), according to the manufacturer’s instructions. Values for total antioxidant capacity were determined by FRAP (ferric reducing antioxidant power assay; [38]), while lipids oxidative damage was assessed by MDA (malondialdehyde; [39]).

### 2.5. Gene Expression by Quantitative PCR

The following steps described, from the RNA extraction to the raw expression data, were carried out by the Unit of Genomics service of the Universidad Complutense de Madrid (UCM, Spain). First, available duodenum tissue samples (~10 to 20 mg) obtained from the birth sampling (48 samples) and slaughterhouse (52 samples; C: 12 males and 9 females, GLN: 15 males and 16 females) were used for total RNA extraction using RNeasy PowerLyzer Tissue and Cells isolation kit (Qiagen, Hilden, Germany) and the TyssueLyser LT equipment (Qiagen), following the manufacturer’s recommendations. The obtained RNA was quantified using NanoDrop ONE (NanoDrop Technologies, Wilmington, DE, USA), and the RNA quality was assessed with an Agilent bioanalyzer device (Agilent Technologies, Palo Alto, CA, USA). Moreover, 2.2 µg of obtained RNA were treated with RNAse-Free DNAse Set (Qiagen), following the manufacturer’s instructions. Second, the synthesis of cDNA was carried out with High Capacity RNA-to-cDNA kit (Applied Biosystems, Foster City, CA, USA) using 9 µl of RNA treated with con DNAse I (~1 µg of total RNA) in a total volume of 20 µL containing, following the supplier’s instructions. Nine target genes related to mechanistic target of rapamycin (mTOR) pathway (linked to Gln; *EIF4EBP1*, *HIFIA*, *MTOR*, *PPARG*, *RPS6*, *RPS6KB1*, *RPTOR*, and *SREBF1* [40,41]) and intestinal tight junctions (*OCLN*; Occludin) and two reference genes (*ACTB* and *GAPDH*) were analyzed. Primer pairs were designed using Universal ProbeLibrary Assay Design Center (Roche Life Science, Basel, Switzerland) from the available ENSEMBL sequences and covered different exons to assure the cDNA amplification (Table S2). Third, the transcript quantification was performed using Power SYBRGreen PCR Master Mix (Applied Biosystems, Warrington, UK) in a QuantStudio 12 K Flex (Applied Biosystems). The qPCR reactions were prepared in a total volume of 10 µL containing 2.5 µL of cDNA (1/10 dilution) and forward and reverse primers (concentration of 300 nM), following the manufacturer’s recommendations. All points and samples were duplicated as technical replicates, and mixes without cDNA were used as negative controls. Cycling conditions were 95 °C for 10 min, followed by 40 cycles of 95 °C (15 s) and 60 °C (1 min). Data were extracted with the QuantStudio 12 K Flex software v1.2.2 (Applied Biosystems).

For statistical analysis of gene expression, the influence of maternal treatment and BIW classification were analyzed with a linear model fitting factors and their interactions as fixed effects with sow (at birth) as a random effect. The method employed for the statistical analysis of the gene expression data [42] simultaneously analyzed the Cp values for the target and endogenous genes using a linear mixed model. The model used and posterior calculations were performed as previously described by our group [43]. Treatment and sexes were assessed at both ages, but BIW was only assessed at birth. The adjusted *p*-values are indicated as *q*-values.

### 2.6. Fat Content and Fatty Acid Composition of Diets and Tissue Samples

The one-step procedure [44] was used for the extraction and methylation of the diet fatty acids (FA). Gas chromatography (Hewlett Packard HP-6890, Palo Alto, CA, USA) was used to identify fatty acid methyl esters by a flame ionization detector and a capillary column (HP-Innowax, 30 m × 0.32 mm i.d., and 0.25 µm polyethylene glycol-film thickness; [45]).

The lipids from the brain, intramuscular fat (IMF) at the LD muscle, and liver fat were extracted [46]. Fat content was expressed as a percentage (%) of dry matter (DM). Afterward, total lipids at IMF and liver fat were separated into the neutral lipid (NL; in fat storage such as triglycerides) and polar lipid fractions (PL; in cell membranes such as phospholipids; [47]). Subcutaneous fat was individually analyzed in outer and inner layers. Extracts were methylated [48] and analyzed using protocols developed at our laboratory [45]. The individual FA percentages for saturated, monounsaturated, and polyunsaturated FA (SFA, MUFA, and PUFA) were calculated. Total n-3, total n-6 FA, the Σn-6/Σn-3 ratio, and the unsaturated index (UI) were also calculated [49]. The activity of stearoyl-CoA desaturase enzyme 1 (SCD1) was estimated as C18:1/C18:0 and MUFA/SFA ratios (desaturation indexes; [50]).

### 2.7. Statistical Analysis

Data were analyzed by the SAS version 9.4 (Statistical Analysis System Institute Inc., Cary, NC, USA) to assess the maternal treatment effect (C vs. GLN) and its interactions with sex (female vs. male) and BIW (LBIW vs. NBIW). Dependent variables were assessed using two-way ANOVA in a general linear model, including maternal treatment and sex. Except at birth and weaning, when the BIW classification was also added to the model (three-way ANOVA) and all maternal treatment interactions. No significant triple and double interactions were removed from models, and significant interactions were studied individually. Changes over time in body weight, backfat depth, and meat color traits were assessed using a repeated measures test with the Greenhouse–Geisser correction. Reproductive data and birth data were analyzed by parity and maternal treatment, and chi-square was used to assess the percentage of LBIW piglets and percentages of parity. Sow was used as a random effect in the birth and weaning analysis to consider the common maternal environment. Litter size was categorized and used as a random effect for birth data [10]. For performance parameters, the respective age was used as a covariate. Finally, pig was the experimental unit for all variables studied except for the reproductive data, where sow was the unit. Results were expressed as mean ± SE. Statistical significance was accepted from *p* < 0.05 and statistical trend was defined as 0.05 < *p* < 0.1.

## 3. Results

### 3.1. Gilts and Sows

Differences in the number of total piglets born (C: 6.7 ± 0.5, GLN: 7.1 ± 0.3 piglets) and piglets born alive (C: 6.4 ± 0.5, GLN: 6.7 ± 0.3 piglets) were not statistically significant between maternal treatments, and neither was for the LBIW piglet proportion at birth (C: 11.7%, GLN: 14.2% of LBIW piglets). Birth weight mean per litter (C: 1.38 ± 0.04, GLN: 1.31 ± 0.03 kg) and its measures of BIW variation (SD, C: 0.19 ± 0.02, GLN: 0.20 ± 0.01 kg; CoV, C: 14.3 ± 1.5, GLN: 15.6 ± 1.1%) were also not different between treatments. Among the rest of the body measures (Table S3), the mean of trunk length was greater in the control litters than in the treated ones (C: 23.8 ± 0.3, GLN: 22.9 ± 0.2 cm; *p* < 0.05), and there was a trend towards a similar difference in the head length (C: 12.8 ± 0.09, GLN: 12.0 ± 0.07 cm; *p* = 0.06). No significant interactions between maternal treatment and sow parity were found, although there were differences by parity (Table S3).

### 3.2. Offspring at Birth

#### 3.2.1. Body Measures and Composition

There were no differences in birth weight (C: 1.32 ± 0.02, GLN: 1.30 ± 0.02 kg) between control and treated alive piglets. Only head (C: 12.11 ± 0.05, GLN: 11.95 ± 0.04 cm; *p* < 0.005) and trunk lengths (C: 23.5 ± 0.2, GLN: 22.9.6 ± 0.1 cm; *p* < 0.05) showed differences by maternal treatment. Control LBIW piglets also had a longer head than treated LBIW ones (C LBIW: 11.4 ± 0.05, GLN LBIW: 11.04 ± 0.04 cm; *p* < 0.05). Regarding viscerae, lungs were heavier in control than in treated newborns (Table 1; *p* < 0.05). Treated newborns showed lighter carcass (*p* = 0.08) and greater relative weights of the liver (*p* = 0.06) and total viscerae (*p* = 0.05) to body weight than control ones. Triple interactions and interactions between sex and maternal treatment were not significant, while the BIW affected almost all variables (Tables S4 and S5).

#### 3.2.2. Plasma Parameters

Regarding the AA plasma levels, Gln was higher in treated newborns than in control ones (Table 1; *p* < 0.05), without significant maternal treatment interactions with BIW or sex. Treated newborns also had greater concentrations of alanine (Ala, Table 1; *p* < 0.005), asparagine (Asn; *p* < 0.05), glycine (Gly; *p* < 0.01), histidine (His; *p* < 0.05), proline (Pro; *p* < 0.05), serine (Ser; *p* < 0.005), and valine (Val; *p* < 0.05) than controls. There was also a trend for higher concentrations of isoleucine (Ile; *p* = 0.07) and tryptophan (Trp; *p* = 0.06). In addition, treated LBIW newborns showed a greater amount of Ala (C: 40.3 ± 6.4, GLN: 77.4 ± 10.8 mg/L; *p* < 0.001) and arginine (Arg, C: 13.6 ± 3.0, GLN: 27.8 ± 5.5 mg/L; *p* < 0.005) than control LBIW ones. The remaining AAs were only affected by the BIW (Table S5) with no interactions with sex. No effect of maternal treatment was observed on glucose and lipid metabolism. The assessment of lipid oxidative damage showed a trend of greater values in control newborns than in treated ones (*p* = 0.08), but similar values of the antioxidant capacity. There were no significant triple interactions nor between sex and maternal treatment (Table S5).

#### 3.2.3. Gene Expression

**Figure 1:**
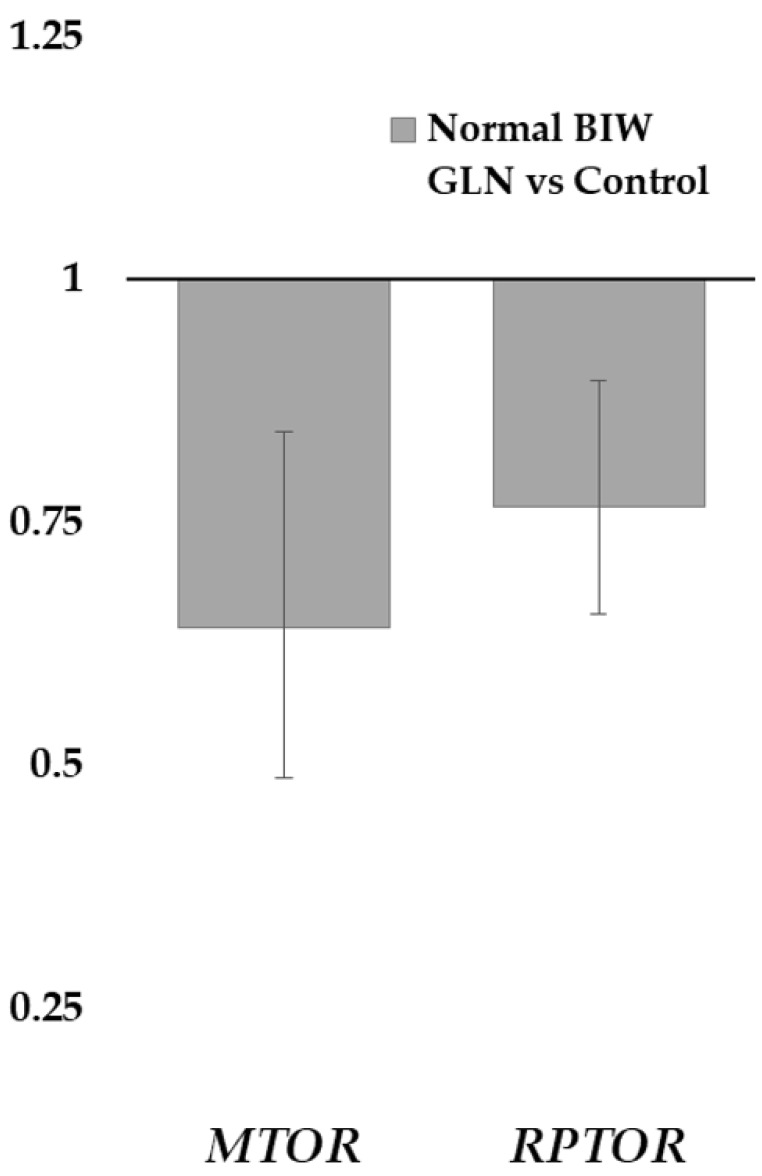
Fold-change (FC) ratios of *MTOR* and *RPTOR* genes with significant differences between maternal treatments within normal birth-weight (BIW) newborns. FC values <1 indicate higher expression in the first group. The error lines indicate the standard errors. GLN: group treated with L-glutamine.

No differences in the expression of target genes were found between maternal treatment groups (Table 2). However, treated NBIW newborns had higher *RPTOR* (Figure 1; *q* < 0.05) and *MTOR* (*q* < 0.05) expression than control NBIW ones. For the *MTOR* gene, there were also different expression levels between BIW groups within the control group (FC: 1.69, *q* < 0.01), being higher in NBIW neonates, but not in the treated group. Further information about gene expression results is shown in Table S6.

#### 3.2.4. Fatty Acid Composition of Brain, Liver, and Muscle Tissues

##### Maternal Treatment Effects

Maternal treatment directly affected the polar lipid fractions in the brain (Table S7), muscle (Table S8), and liver (Table S9). Concentrations of Σn-3 FA in brain and muscle were greater in treated than in control newborns (*p* < 0.05). In concordance, the Σn-6/Σn-3 FA ratio (*p* < 0.05) was also lower in treated newborns in muscle. On the contrary, the liver fat content was lower in treated than in control newborns (Table 3; *p* < 0.01). In the liver, treated newborns also showed a greater desaturation index (Table 3; *p* < 0.01) than controls, mainly influenced by C18:1-n9 FA (oleic acid, Table S9), and concentrations of MUFA and PUFA were also affected (Table 3; *p* = 0.07 for both).

##### Maternal Treatment Interactions

Regarding the LBIW group, control newborns showed greater Σn-6 FA values than treated ones in the polar lipid fraction of the brain (Table 4; *p* < 0.01) with a higher Σn-6/Σn-3 FA ratio (*p* < 0.05) and a lower C18:1 FA/C18:0 FA index (*p* < 0.05). Control LBIW newborns also had lower unsaturated indexes (*p* < 0.01) and greater SFA amounts (*p* < 0.05) than treated LBIW ones in the neutral lipid fractions of muscle and higher SFA amounts (*p* < 0.05) in the neutral lipid fractions of the brain.

Differences between control LBIW and NBIW newborns were found in the neutral and polar lipid fractions of the brain and the neutral fraction of the liver and muscle (Table 4), but not between treated LBIW and NBIW ones. Unsaturation indexes (MUFA/SFA or C18:1 FA/C18:0 FA) were higher in control NBIW newborns than in their LBIW counterparts in previously named lipid fractions (*p* < 0.01 for all, except in the neutral lipid fraction of the liver, *p* < 0.05). Saturated FA values were greater in the brain and muscle neutral lipid fraction of control LBIW neonates than of control NBIW ones (*p* < 0.005, for both), and MUFA values in the brain polar lipid fraction were lower (*p* < 0.005). Control NBIW newborns had greater Σn-6 FA concentrations (*p* < 0.0001) than their LBIW counterparts in the polar lipid fraction of the brain, and with higher values of the Σn-6/Σn-3 FA ratio (*p* < 0.005).

### 3.3. Postnatal Development

At weaning, there were no differences in ADWG values, body weight, or body measures considering maternal treatment or its interactions (Table S10), except for a longer biparietal diameter in control piglets (6.60 ± 0.05 vs. 6.48 ± 0.04 cm, *p* < 0.05). However, treated males (0.50 ± 0.02 cm, *p* < 0.0001) and control females (0.46 ± 0.02 cm, *p* < 0.0001) showed thicker backfat depth than treated females (0.41 ± 0.02 cm). There were also differences related to backfat depth between groups of maternal treatment affected by sex at 215 days-old, but not in its evolution during the postnatal development. Control males had thinner total backfat depth than treated males (2.11 ± 0.1 vs. 2.36 ± 0.07 cm, *p* < 0.05), mainly due to a smaller inner layer (1.05 ± 0.07 vs. 1.23 ± 0.05 cm, *p* < 0.05). Control males also showed thinner total backfat depth than control females (2.57 ± 0.11 cm, *p* < 0.005) and also in both layers (Inner, 1.32 ± 0.08 cm, *p* < 0.05; Outer, 1.06 ± 0.06 vs. 1.25 ± 0.05 cm, *p* < 0.01). At 215 days-old, there was no effect of maternal treatment on plasma parameters (glucose and lipid metabolism).

Throughout their total postnatal period, treated pigs (ADWG: 516 ± 4 g/day; *p* < 0.005) grew slower than controls (536 ± 5 g/day), particularly the females (C: 544 ± 8 GLN: 511 ± 7 g/day; *p* < 0.005). The evolution of body weight was also different between groups of maternal treatment (*p* < 0.005), especially after weaning (*p* < 0.005). At the slaughterhouse, treated pigs were lighter than controls (C: 144.1 ± 1.5 GLN: 138.6 ± 1.2 kg; *p* < 0.005). In the control group, males reached their market weight sooner (Market age 264 ± 1.3 days; *p* < 0.005) than females (270 ± 1.7 days).

#### 3.3.1. Carcass Traits and Gene Expression at the Slaughterhouse

Control females showed longer carcasses than control males (92.0 ± 0.6 vs. 89.8 ± 0.5 cm, *p* < 0.05) and treated females (89.4 ± 0.5 cm, *p* < 0.005). However, no differences were found in carcass weight or yield nor in the total backfat depth, neither in muscle drip-loss, nor in the pH values between maternal treatment groups, nor by interaction with sex (Table S10). On the other hand, the expression of target genes did not differ by maternal treatment (Table 5).

#### 3.3.2. Fatty Acid Composition of Muscle, Backfat, and Liver Tissues at the Slaughterhouse

##### Maternal Treatment Effects

The FA composition of muscle (Table S12), backfat (Table S13), and liver (Table S14) showed some differences by maternal treatment. Regarding the FA composition of LD muscle, control pigs showed greater values of SFA, PUFA (both Σn-6 and Σn-3 FA), and of both desaturation indexes than treated ones in the neutral lipid fraction (Table 6, *p* < 0.05 for all) and a trend to lower MUFA concentrations (*p* = 0.05).

##### Maternal Treatment Interactions

The remaining differences in FA composition were conditioned by sex (Table 7). In the polar lipid fraction of the LD muscle, control females showed the lowest value of MUFA (*p* < 0.05 for all) and the highest values of PUFA (and Σn-6 FA, *p* < 0.05 for all) than the rest of the groups. They also had lower SFA concentrations than treated females and control males (*p* < 0.05 for all). However, the lowest values of Σn-3 FA (*p* < 0.001 for all) and the greatest Σn-6/Σn-3 FA ratio (*p* < 0.001 for all) belonged to treated females. In the FA profile of backfat, only a desaturation index (C18:1/C18:0) of the inner layer was greater in control males than in the rest of the groups (vs. control females and treated males, *p* < 0.05; and vs. treated females, *p* = 0.06). Finally, treated females showed lower concentrations of SFA (Table 7, *p* < 0.05) and greater PUFA values (only Σn-6 FA; *p* < 0.05 for both) than control females in the liver polar lipid fraction. On the other hand, treated females also showed a lower SFA concentration in the liver neutral lipid fraction than treated males (*p* < 0.05).

## 4. Discussion

The industry of traditional swine breeds is currently increasing its census, production, and economic influence. Batch homogeneity is one of the main production challenges, so the search for useful solutions for its improvement from birth is critical. Thus, testing feed supplements showing positive results in other swine breeds, such as Gln, is required, particularly considering the metabolic differences between traditional and selected swine breeds [28,29,30,31,32,33,34]. Glutamine is one of the most interesting AA in fetuses and adults because it is one of the most used AA by pig enterocytes as an energy source and in all tissues for nucleic acid synthesis [18,19]. The current study is not only important because it is the first one focused on the effect of prenatal Gln supplementation on traditional breeds, but because it is also the first trial of a prenatal supplementation of any AA in this kind of swine breeds.

Among the valuable data of this study is the first assessment of plasma AA levels in newborns of the Iberian breed. Some AA levels (i.e., Gln, Ala, and Asn) were greater in treated newborns. Moreover, the BIW was also studied, and the treated LBIW group showed higher Arg than the corresponding control group. The transfer of Gln to fetal blood is critical for maximizing fetal growth by synthesizing other AA, such as Glu, Asp, and Asn [51]. In a previous study, the maternal concentrations of Gln in Meishan gilts (Pa1, nulliparous), a highly prolific breed with a remarkable homogeneity in the offspring, was higher than in Large White × Landrace gilts [52,53,54,55]. The Meishan newborns also had greater Gln, Ala, and Asp concentrations, similarly to Gln supplemented newborns in a previous study, which showed higher Gln values [27,55]. However, only the proportional weight of viscerae was higher in our treated newborns related to prenatal body growth. Glutamine is recognized as a nutritionally essential AA for gestating, especially after the early gestation period and in gilts, so more improvements in litters from gilts were expected [15,56]. On the contrary, the lack of effects on the development of litters from sows supplemented with Gln has been previously described in selected swine breeds [57,58]. However, the promising potential of the Gln maternal supplementation to reduce negative effects of the IUGR process, according to scientific literature [15,19,27,59,60], led to expect more effects than those found under our conditions. Nevertheless, future studies with larger sample size, particularly gilts, could increase the detection power of productive effects, based on the result of previous studies carried out in other swine breeds.

Regarding the FA profile of different tissues, there were significant effects of the maternal treatment at birth. First, the control group showed differences between LBIW and NBIW newborns, but not in the treated group. Thus, the FA composition at birth would be more homogeneous in newborns from pregnancies supplemented with Gln. Second, treated newborns showed greater amounts of Σn-3 FA in brain and muscle cellular membranes, related to a protective effect because of the improvement of pro-/anti-inflammatory status and the reduction of pathological risks [61,62]. Finally, the treated group had a higher amount of C18:1 FA in the liver, which was previously found in Meishan newborns [54]. This result could be associated with a possible adaptation to increase the survival capacity [54], although it was not possible to corroborate it with our results.

On the other hand, the supplementation of Gln also activates, directly and indirectly, the protein synthesis in fetal skeletal muscle and the mTOR signaling pathway, linked to cell growth and proliferation [19,23,40,63]. Our study has shown higher expression in components of the mTOR protein complex 1 (mTORC1; *MTOR* and *RPTOR* [raptor]) in treated NBIW newborns than in their counterparts of the control group. The mTORC1 is related to the regulation of protein and lipid synthesis, autophagy, and energy metabolism, but none of its downstream genes studied showed differences between treatment groups [40,41,63]. This finding would consist of the lack of evidence of greater prenatal growth in supplemented pregnancies. Furthermore, differences in the *MTOR* expression between LBIW and NBIW of the control group, but not in the treated group, were also found. Thus, treated newborns showed, as in the FA profile, more homogeneity than the control group. Finally, the expression related to intestinal tight junctions was also not different by treatment, although a previous study found benefits from the Gln supplementation in LBIW and NBIW [27]. So, physiological mechanisms related to Gln could improve prenatal development that would improve productive parameters at birth. However, 1% Gln supplementation in Iberian gilts and sows under our conditions seems not to be enough to trigger it.

The current study is the first to show the offspring development after weaning in pigs from pregnancies supplemented with Gln. Although it is always a challenge to find medium or long-term effects of maternal supplementations on the offspring, this valuable data allows us to assess the possibility of physiological or cellular changes with postnatal effects due to the prenatal Gln supplementation. However, no beneficial effects of the maternal treatment were found at weaning, at 215 days-old, or at the slaughterhouse under our conditions. Furthermore, some differences in backfat depth, carcass traits, and tissue FA profiles were affected by sex, mainly in females, although no sex-related effect was found at birth. Nevertheless, sex is a well-known important factor in swine production and, especially, in the Iberian breed [10,64,65,66].

## 5. Conclusions

In the current study, the Gln supplementation at 1% after Day 35 of gestation improved the plasma AA levels, the FA profile of cellular membranes in several tissues, and the gene expression of mTORC1 in Iberian newborns. However, these findings have not turned into advantageous effects on productive traits at birth nor later periods in the litters of gilts or sows under our conditions. So, further research is needed to deepen the knowledge of the parameters and molecular pathways affected by the Gln supplementation as a nutritional strategy at the sow level. Furthermore, according to our results, differences at the productive or physiological level with swine breeds previously tested should be considered before directly implementing nutritional strategies in other breeds.

## Figures and Tables

**Table 1:** Significant differences between control and treated (GLN) newborns at birth. Mean ± SE.

	Control	*n* = 24	GLN	*n* = 24	*p*-Value
**Plasma amino acids (mg/L)**					
GLN	90.20	±6.92	110.60	±7.36	0.03
ALA	48.65	±4.31	70.53	±5.97	0.004
ASN	15.01	±1.34	19.21	±1.79	0.03
GLY	49.26	±3.75	66.64	±4.51	0.006
HIS	21.58	±1.96	30.14	±2.74	0.01
PRO	66.4	±12.8	100.5	±11.3	0.02
SER	21.43	±1.79	31.32	±2.75	0.004
VAL	33.82	±3.06	43.24	±3.39	0.03
ILE	6.40	±0.83	9.45	±1.49	t
TRP	9.95	±0.43	11.30	±0.61	t
**Body composition**					
Lung W (g)	21.42	±1.51	18.67	±1.53	0.04
Liver W/Body W (%)	2.51	±0.09	2.84	±0.16	t
Carcass W (g)	689.5	±57.0	600.5	±56.0	t
All viscera W/Body W (%)	14.03	±0.61	15.42	±0.46	t
**Plasma parameters**					
MDA (µmol/L)	2.64	±0.46	1.61	±0.29	t

W = weight, Amino acids (ALA = Alanine, ASN = Asparagine, GLN = Glutamine, GLY = Glycine, HIS = Histidine, ILE = Isoleucine, PRO = Proline, SER = Serine, TRP = Tryptophan, VAL = Valine), MDA = malondialdehyde, SE = standard error, t = 0.1 > *p* > 0.05.

**Table 2:** Expression of target genes in control and treated (GLN) newborns at birth.

Gene	FC (GLN-C)	95% CI	*p*-Value/*q*-Value
*EIF4EBP1*	0.82	0.50–1.33	ns/ns
*HIF1A*	1.15	0.35–3.84	ns/ns
*MTOR*	0.85	0.70–1.03	0.08/ns
*OCLN*	0.75	0.46–1.22	ns/ns
*PPARG*	0.92	0.57–1.50	ns/ns
*RPS6*	0.83	0.67–1.02	0.07/ns
*RPS6KB1*	0.83	0.71–0.97	0.02/ns
*RPTOR*	0.89	0.79–0.99	0.03/ns
*SREBF1*	0.89	0.67–1.18	ns/ns

FC = Fold change, CI = Confident interval, ns = not significant.

**Table 3:** Significant differences in tissue fatty acid (FA) compositions between control and treated (GLN) newborns at birth. Mean ± SE (g/100 g total FA).

	Control	*n* = 24	GLN	*n* = 24	*p*-Value
**Liver, Polar lipids**					
Fat (% dry matter)	18.44	±0.67	15.73	±0.69	0.009
MUFA	25.02	±0.45	26.36	±0.52	t
PUFA	34.3	±0.3	33.3	±0.4	t
C18:1/C18:0	1.04	±0.03	1.14	±0.04	0.04
**Brain, Polar lipids**					
PUFA Σn-3	8.53	±0.25	9.04	±0.13	0.04
***LD* muscle, Polar lipids**					
PUFA Σn-3	3.53	±0.07	3.78	±0.07	0.02
Σn-6/Σn-3	6.62	±0.12	6.19	±0.13	0.02

MUFA = sum of monounsaturated FA, PUFA = sum of polyunsaturated FA, *LD* = *Longissimus dorsi*, SE = standard error, t = 0.1 > *p* > 0.05.

**Table 4:** Significant differences in tissue fatty acid (FA) compositions at birth between newborns with low and normal birth-weight (LBIW and NBIW) from both control (C) and treated (GLN) groups. Mean ± SE (g/100 g total FA).

	C NBIW *n* = 12	C LBIW *n* = 12	GLN LBIW *n* = 12
**Liver, Neutral lipids**			
Σn-6/Σn-3	5.39 ± 0.25	4.43 ± 0.24 ^T^	
C18:1/C18:0	4.33 ± 0.32	3.23 ± 0.22 ^A^	
**Brain, Neutral lipids**			
SFA	45.61 ± 0.28 ^C^	48.25 ± 0.34 ^C,A^	47.08 ± 0.39 ^A^
MUFA/SFA	0.57 ± 0.01	0.51 ± 0.01 ^B^	
**Brain, Polar lipids**			
MUFA	26.02 ± 0.24	25.10 ± 0.36 ^C^	
PUFA Σn-6	16.68 ± 0.14 ^D^	17.80 ± 0.24 ^D,B^	17.19 ± 0.07 ^B^
Σn-6/Σn-3	1.82 ± 0.04 ^C^	2.38 ± 0.22 ^C,A^	1.98 ± 0.05 ^A^
MUFA/SFA	0.55 ± 0.01	0.52 ± 0.01 ^B^	
C18:1/C18:0	1.20 ± 0.02 ^B^	1.15 ± 0.01 ^B,A^	1.20 ± 0.01 ^A^
***LD* muscle, Neutral lipids**			
SFA	43.44 ± 0.40 ^C^	45.92 ± 0.63 ^C^	43.46 ± 0.49 ^A^
MUFA/SFA	0.98 ± 0.02 ^B^	0.89 ± 0.03 ^B,B^	0.98 ± 0.02 ^B^

NBIW = Normal BIW, LBIW = Low BIW, SFA = sum of saturated FA, MUFA = sum of monounsaturated FA, PUFA = sum of polyunsaturated FA, *LD* = Longissimus dorsi, SE = standard error, Superscritps: T = 0.1 > *p* > 0.05; ^A^ = *p* < 0.05; ^B^ = *p* < 0.01; ^C^ = *p* < 0.005; ^D^ = *p* < 0.0001.

**Table 5:** Expression of target genes in control (C) and treated (GLN) pigs at the slaughterhouse.

Gene	FC (GLN-C)	95% CI	*p*- & *q*-Values
*EIF4EBP1*	0.99	0.79–1.24	ns
*HIF1A*	1.03	0.84–1.27	ns
*MTOR*	1.07	0.83–1.38	ns
*OCLN*	1.07	0.85–1.36	ns
*PPARG*	1.24	0.93–1.66	ns
*RPS6*	1.12	0.87–1.43	ns
*RPS6KB1*	1.36	0.71–2.58	ns
*RPTOR*	1.02	0.80–1.31	ns
*SREBF1*	0.99	0.77–1.27	ns

FC = Fold change, CI = Confident interval, ns = not significant.

**Table 6:** Significant differences in the fatty acid (FA) composition of the polar lipid fraction of LD muscle between control and treated (GLN) pigs at the slaughterhouse. LSmean ± SE (g/100 g total FA).

	Control	*n* = 54	GLN	*n* = 79	*p*-Value
***LD* muscle, Neutral lipids**					
SFA	38.62	±0.59	39.67	±0.33	0.04
MUFA	58.66	±0.56	57.76	±0.30	t
PUFA	2.72	±0.06	2.57	±0.05	0.02
Σn-3	0.22	±0.01	0.21	±0.00	0.03
Σn-6	2.34	±0.05	2.21	±0.04	0.02
MUFA/SFA	1.55	±0.04	1.47	±0.02	0.02
C18:1/C18:0	4.71	±0.15	4.47	±0.09	0.03

SFA = sum of saturated FA, MUFA = sum of monounsaturated FA, PUFA = sum of polyunsaturated FA, *LD* = Longissimus dorsi, SE = standard error, t = 0.1 > *p* > 0.05.

**Table 7:** Significant differences in tissue fatty acid (FA) compositions between control (C) and treated (GLN) pigs of both sexes at the slaughterhouse. LSmean ± SE (g/100 g total FA) of C and GLN females (Fem) and males (Mal).

	C Fem *n* = 21	GLN Fem *n* = 30	C Mal *n* = 33	GLN Mal *n* = 49
***LD* muscle, Polar lipids**				
SFA	31.49 ± 0.30 ^A,A^	32.31 ± 0.25 ^A^	32.24 ± 0.24 ^A^	
MUFA	20.57 ± 0.31 ^A,A,B^	21.60 ± 0.26 ^A^	21.58 ± 0.24 ^A^	21.60 ± 0.20 ^B^
PUFA	47.94 ± 0.39 ^D,D,B^	46.09 ± 0.31 ^D^	46.17 ± 0.33 ^D^	46.69 ± 0.26 ^B^
Σn-3	2.80 ± 0.08 ^E^	2.34 ± 0.07 ^E,D,E^	2.71 ± 0.07 ^E^	2.64 ± 0.05 ^D^
Σn-6	44.50 ± 0.23 ^B,B,A^	43.10 ± 0.34 ^B^	42.79 ± 0.37 ^B^	43.38 ± 0.24 ^A^
Σn-6/Σn-3	15.97 ± 0.86 ^D^	19.93 ± 0.73 ^D,D,E^	15.99 ± 0.69 ^E^	16.79 ± 0.56 ^D^
**Backfat, Inner layer**				
C18:1/C18:0	3.36 ± 0.14 ^C^	3.60 ± 0.12 ^T^	3.91 ± 0.11 ^C,T,A^	3.62 ± 0.09 ^A^
**Liver, Neutral lipids**				
SFA		45.66 ± 0.56		47.35 ± 0.44 ^A^
**Liver, Polar lipids**				
SFA	51.73 ± 0.45	50.25 ± 0.36 ^A^		
PUFA	25.80 ± 0.93	28.20 ± 0.74 ^A^		
Σn-6	23.00 ± 0.81	25.21 ± 0.64 ^A^		

SFA = sum of saturated FA, MUFA = sum of monounsaturated FA, PUFA = sum of polyunsaturated FA, *LD* = Longissimus dorsi, SE = standard error, Superscritps: T = 0.1 > *p* > 0.05; ^A^ = *p*< 0.005; ^B^ = *p* < 0.01; ^C^ = *p* < 0.005; ^D^ = *p* < 0.001; ^E^ = *p* < 0.0005.

## Data Availability

Data available in Supplementary Materials and upon request to the corresponding author.

## Supplementary Materials

The following are available online at https://www.mdpi.com/2076-2615/11/3/903/s1, Table S1: Calculated analysis (g/kg, dry-matter basis) and fatty acid composition of the diets, Table S2: Primer design for qPCR and PCR efficiencies, Table S3: Birth data and the mean per sow of body measures of offspring between control (C) and treated (GLN) groups, Table S4: Body measures of piglets born alive at birth, Table S5. Body measures and plasma parameters of control (C) and treated (GLN) slaughtered newborns at birth, Table S6. Fatty acids (FA) composition of the brain (g/100 g total FA) of control (C) and treated (GLN) slaughtered newborns at birth, Table S7. Fatty acids (FA) composition of the liver (g/100 g total FA) of control (C) and treated (GLN) slaughtered newborns at birth, Table S8. Fatty acids (FA) composition of the Longissimus dorsi muscle (g/100 g total FA) of control (C) and treated (GLN) slaughtered newborns at birth, Table S9. The candidate gene expression from control and treated (GLN) slaughtered newborns at birth, Table S10. Postnatal development and final traits of control (C) and treated (GLN) slaughtered pigs, Table S11. The candidate gene expression from control (C) and treated (GLN) pigs at the slaughterhouse, Table S12. Fatty acids (FA) composition of the Longissimus dorsi muscle (g/100 g total FA) of control (C) and treated (GLN) pigs at the slaughterhouse, Table S13. Fatty acids (FA) composition of the backfat (g/100 g total FA) of control (C) and treated (GLN) pigs at the slaughterhouse, Table S14. Fatty acids (FA) composition of the liver (g/100 g total FA) of control (C) and treated (GLN) pigs at the slaughterhouse.
